# The Genetic Base for Peanut Height-Related Traits Revealed by a Meta-Analysis

**DOI:** 10.3390/plants10061058

**Published:** 2021-05-25

**Authors:** Juan Wang, Caixia Yan, Dachuan Shi, Xiaobo Zhao, Cuiling Yuan, Quanxi Sun, Yifei Mou, Haoning Chen, Yuan Li, Chunjuan Li, Shihua Shan

**Affiliations:** 1 Shandong Peanut Research Institute, Qingdao 266100, China; wangjuan_1984@163.com (J.W.); cxyan335@sina.com (C.Y.); zhaoxiaoboqd@126.com (X.Z.); yuancl1982@163.com (C.Y.); squanxi@163.com (Q.S.); yifeimou1123@163.com (Y.M.); chny.312@163.com (H.C.); 2 Qingdao Academy of Agricultural Sciences, Qingdao 266100, China; catchabreak@163.com; 3 Computational Biology and Biological Physics, Astronomy and Theoretical Physics, Lund University, 22100 Lund, Sweden

**Keywords:** peanut, plant height, secondary metabolites, meta-analysis

## Abstract

Peanut (*Arachis hypogaea* L.) is an important oilseed crop worldwide, and peanut height has been shown to be closely related to yield, therefore a better understanding of the genetic base of plant height-related traits may allow us to have better control of crop yield. Plant height-related traits are quantitative traits that are genetically controlled by many genes, and distinct quantitive trait loci (QTLs) may be identified for different peanut accessions/genotypes. In the present study, in order to gain a more complete picture of the genetic base for peanut height-related traits, we first make use of the high quality NGS sequence data for 159 peanut accessions that are available within our research groups, to carry out a GWAS study for searching plant height-related regions. We then perform a literature survey and collect QTLs for two plant height-related traits (Ph: peanut main stem height, and Fbl: the first branch length) from earlier related QTL/GWAS studies in peanut. In total, we find 74 and 21 genomic regions that are, associated with traits Ph and Fbl, respectively. Annotation of these regions found a total of 692 and 229 genes for, respectively, Ph and Fbl, and among those genes, 158 genes are shared. KEGG and GO enrichment analyses of those candidate genes reveal that Ph- and Fbl-associated genes are both enriched in the biosynthesis of secondary metabolites, some basic processes, pathways, or complexes that are supposed to be crucial for plant development and growth.

## 1. Introduction

As one important source of edible oil, the groundnut (*Arachis hypogaea* L.) is widely cultivated across warm temperate, subtropical, and tropical zones [1,2,3], with China being its largest producer and exporter [4]. Nevertheless, there is still a huge market demand for peanuts, which may be met by genetic improvement of its agronomic traits [5,6]. Peanut height has been shown to be closely related to yield, and co-localization of QTLs (Quantitative Trait Loci) for peanut height- and yield-related traits have also been reported [7,8,9,10,11], therefore, understanding the genetic base of plant height-related traits may allow us to have better control of crop yield. The plant height-related traits, such as plant main stem height (Ph), nodes on the main stem (Nsk), and the first branch length (Fbl), are quantitative traits, which are controlled by multiple loci and at the same time influenced by variable environmental factors [7,12]. Therefore, it is very challenging to explicitly address the genetic base of a plant height-related trait, nevertheless, efforts have been made by both QTL mapping as well as GWAS studies in recent years, which move us closer and closer towards this goal [7,9,13,14,15,16,17].

In the current study, we make use of the available NGS data for 159 of the 195 peanut accessions that were generated by our earlier yield-related trait-focused GWAS study [18] to further explore the genetic base of plant height-related traits. These NGS data together with phenotype data on three plant height-related traits that were collected from the same peanut accessions make it possible for us to perform GWAS analyses, from which candidate genomic regions have been identified for two of the studied traits. Meanwhile, we have also collected QTLs, for the same traits, from earlier QTL/GWAS analyses that together represent a high diversity of peanut genotypes. A summary of all the annotated genes within the candidate genomic regions from both the present GWAS and earlier QTL/GWAS analyses have helped us gain a more complete picture of the underlying genetic base for the two height-related traits.

## 2. Results

### 2.1. Characterisation and Distribution of Genetic Variations of the Peanut Genome

**Figure 1:**
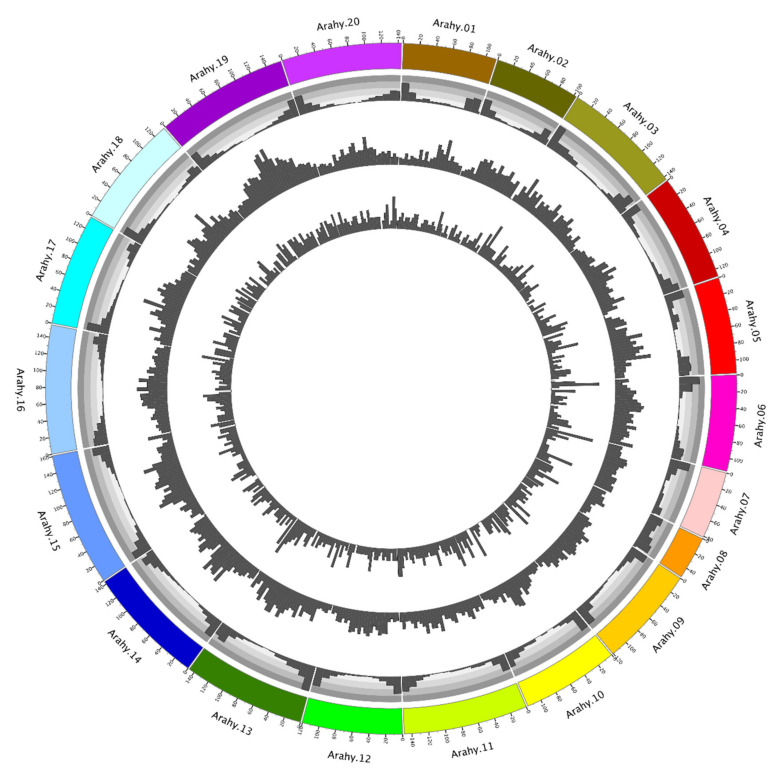
Genome map of peanut. The four layers from the outmost to the innermost, respectively, represent chromosomes, gene density, SNP density, and InDel density.

A total of 15,556 SNPs and 1561 InDels have been identified from the collected peanut genomes (Table S2), leading to a genome-wide SNP density of 6.13 SNPs/Mb, with chromosomes Arahy.08 (4.24 SNPs/Mb) and Arahy.19 (8.77 SNPs/Mb) being, respectively, the sparsest and the densest. Most of the identified SNPs are found at intergenic regions (90.12%), while the exonic, intronic, upstream, and downstream regions only account for 3.05%, 3.78%, and 3.05% of the total SNPs, respectively. Similarly, the genome has a genome-wide InDel density of 0.61 InDels/Mb with chromosomes Arahy.02 (0.39 InDels/Mb) and Arahy.08 (0.91 InDels/Mb) having, respectively, the sparsest and the densest InDels (Figure 1). Most of the identified InDels are also found at intergenic regions (71.68%), while the exonic, intronic, up- and down-stream regions only contain, respectively, 5.06%, 13.84% and 9.42% of the total InDels. The genome-wide transition/transversion (*T*s/*T*v) ratio for the analyzed peanut genome data is 1.81.

### 2.2. Phenotypic Correlation and Heritability for Different Traits

A total of 159 peanut accessions have been phenotyped for their plant main stem height [Ph], the first branch length [Fbl], and the number of nodes on the main stem [Nsk] over three years at three different locations. All traits follow a normal distribution with low values of skew and kurtosis (Supplementary Materials Figures S2–S5). We observe an intermediate level of heritability (*H*^2^: 0.44–0.51) for all three traits, which are positively correlated with each other (*r*: 0.55–0.64) (Table 1 and Table S3; Figure S6).

### 2.3. Genome-Wide Association Study (GWAS) in Peanut

**Figure 2:**
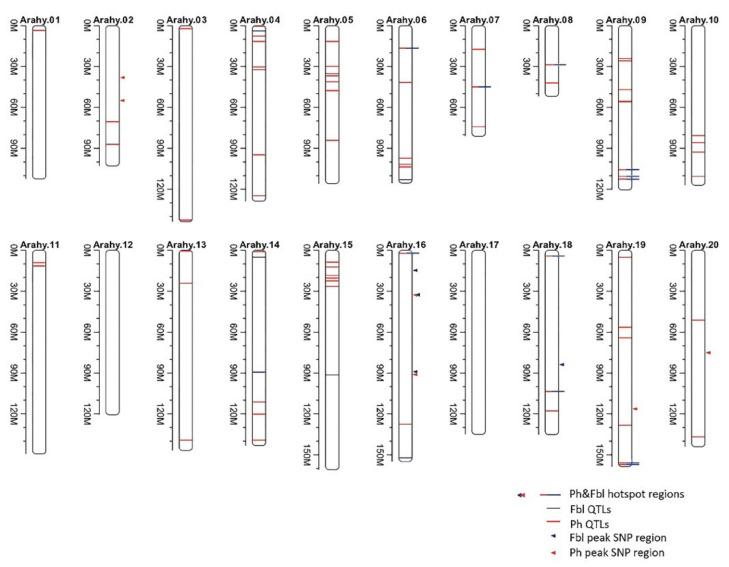
Quantitative trait loci (QTLs) identified to be associated with Ph and/or Fbl by both this and earlier studies. The blue lines represent the QTLs for Fbl identified from earlier studies while red for Ph. The solid triangles point to the peak SNP regions that are identified to be associated with Ph (red) and/or Fbl by the present GWAS analysis.

Genome-wide association analysis of the phenotypically characterized peanut accessions has been conducted to see if any of the acquired SNPs are associated with the studied plant height-related traits. For traits Ph and Fbl, tests with the MLM (K) and MLM (QK) models, especially MLM (QK), produce *p*-values that deviate dramatically less from expectation than the *p*-values using the GLM and GLM (Q) models (QQ plots in Figure S7). However, for MLM (QK), the signal for a significant association is weak, especially the test *p*-values become larger than expected when it goes towards the QQ plot tip, suggesting possible overcorrection of confounding factors by the *Q* and *K* matrices [19]. Nevertheless, under the MLM (QK) model, we are still able to detect significantly associated peak SNPs for the studied traits by a significance level of 10^−5^ (1.1 × 10^-6^ ~ 9.6 × 10^−6^). For Ph, two of the identified associated peak SNPs are located on chromosome Arahy.02, three on Arahy.16, one on each of Arahy.19, and Arahy.20 (Figure 2). For Fbl that has a relatively higher heritability (*H*^2^ = 0.51) comparing to Ph, only four associated peak SNPs have been identified, three from Arahy.16 and one on Arahy.18. It is worth noting that traits Ph and Fb1 share one associated peak SNPs on Arahy.16, Arahy.16:32726692, and even more interestingly, from the candidate genome region that is centered on this shared peak SNP, two genes (arahy.DT3E7E at Arahy.16:32705499-32708164; arahy.AG6W8J at Arahy.16:32708616-32709684) are annotated to be LRR receptor-like kinase. LRR receptor-like kinases are mostly involved in signal transduction and play an essential role in plant development and stress resistance [20,21,22]. No significant association has been found for the trait Nsk.

### 2.4. A Summary of QTLs, for Peanut Ph and Fbl, Identified from Both Present and Earlier Studies

We have performed a thorough literature survey and found twelve QTL/GWAS studies that also looked at the same peanut phenotypic traits as we do though with different sets of peanut accessions (Table S4). These studies, together with the current GWAS study, have identified 74 and 21 genomic regions that are, respectively, associated with traits Ph and Fbl (Table S5; Figure 2). Annotation of these regions found a total of 692 and 229 genes for, respectively, Ph and Fbl, among those genes, 158 genes are shared (Tables S5 and S6).

**Figure 3:**
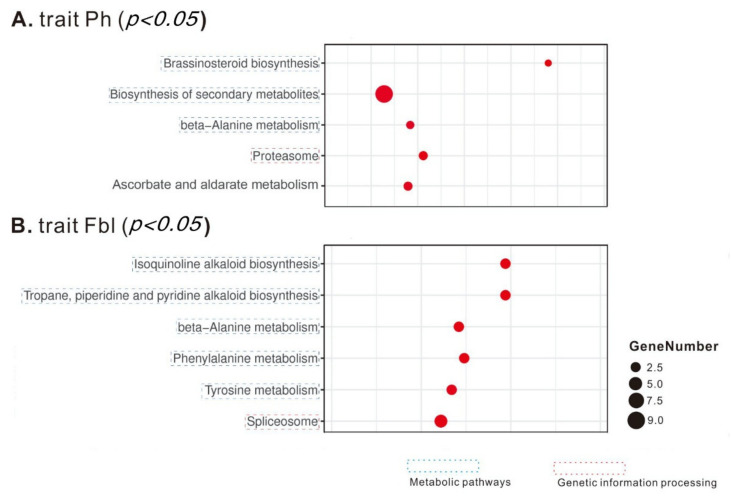
The KEGG pathway enrichment scatter plots of the candidate genes for Ph (**A**) and Fbl (**B**). Only significant pathways are shown here. The number of candidate genes is indicated by the size of a circle.

**Figure 4:**
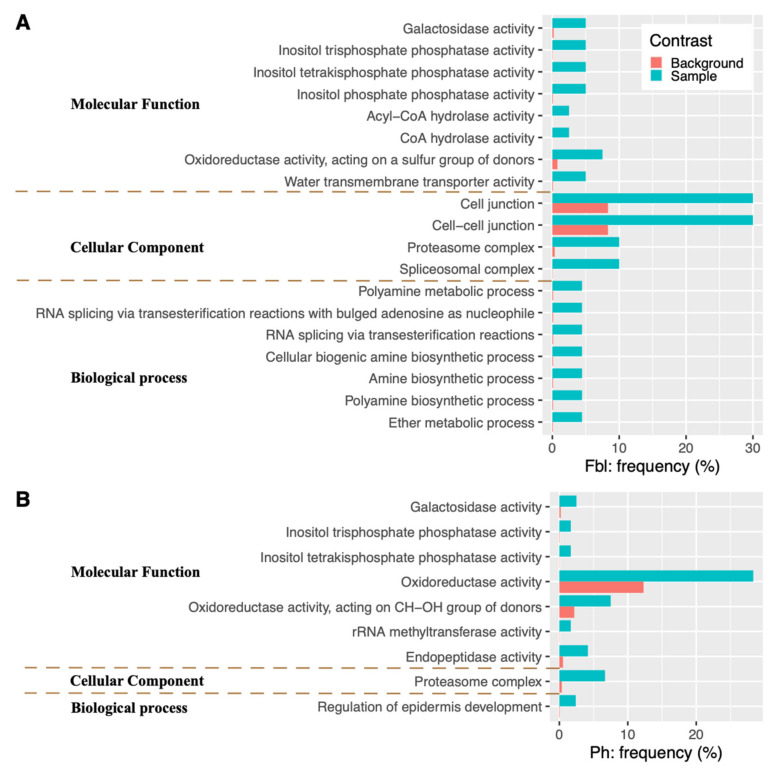
GO terms of candidate genes significantly enriched for Fbl (**A**) and Ph (**B**). The shown gene frequencies for each GO term are either among the candidate genes (blue) or among the background gene database (orange).

KEGG enrichment analysis found that the Ph-associated genes are enriched in four metabolic pathways (Brassinosteroid biosynthesis, Biosynthesis of secondary metabolites, beta-Alanine metabolism, and Ascorbate and aldarate metabolism) as well as one genetic information processing complex (Proteasome) (Figure 3). While GO enrichment analyses show that the candidate genes for Ph are enriched in the proteasome complex (the “Cellular Component” category), in the developmental process of regulating epidermis development (the “Biological Process” category), as well as in several catalytic activities (the “Molecular Function” category): oxidoreductase activity, endopeptidase activity, rRNA methyltransferase activity, oxidoreductase activity (acting on CH-OH group of donors), plus three hydrolase activities (inositol tetrakisphosphate phosphatase activity, inositol trisphosphate phosphatase activity, and galactosidase activity) (Figure 4).

For Fbl, its associated genes are over-represented in one KEGG genetic information processing complex (Spliceosome) and several KEGG metabolic pathways: Biosynthesis of secondary metabolites (Isoquinoline alkaloid biosynthesis; Tropane, piperidine, and pyridine alkaloid biosynthesis), Amino acid metabolism (Phenylalanine metabolism, and Tyrosine metabolism), and beta-Alanine metabolism (Figure 3). In the GO enrichment analysis of the candidate genes for Fbl, among the biological process category, seven metabolic processes (ether metabolic process, polyamine biosynthetic process, amine biosynthetic process, cellular biogenic amine biosynthetic process, RNA splicing via transesterification reactions, RNA splicing via transesterification reactions with bulged adenosine as nucleophile, and polyamine metabolic process) are the most representative groups (Figure 4). For molecular function, the top eight groups include one transporter activity (water transmembrane transporter activity), and seven catalytic activities: oxidoreductase activity (acting on a sulfur group of donors), plus six hydrolase activities (inositol tetrakisphosphate phosphatase activity, inositol trisphosphate phosphatase activity, inositol phosphate phosphatase activity, galactosidase activity, CoA hydrolase activity, and acyl-CoA hydrolase activity). In the cellular components, the highly representative groups are spliceosomal complex, proteasome complex, cell-cell junction, and cell junction (Figure 4).

## 3. Discussion

The cultivated peanut is an important oilseed crop and it is widely cultivated across warm temperate, subtropical, and tropical areas [1,3]. Peanut originated in South America, and from there it has been spread around the world, and in order to adapt to its new and diverse environments, peanut evolved both genetically and phenotypically. In the current study, we have studied 159 peanut accessions, which encompass rich genetic variations by including most of the Chinese peanut landraces [23]. Based on the collected genotypic and phenotypic data, genome-wide association analyses have been performed. The identified candidate genomic regions together with those from earlier QTL analyses are combined in the present study in order to help gain a more comprehensive understanding of the underlying genetic basis for plant height-related traits.

The plant height-related traits are quantitative traits that depend on the cumulative actions of multiple genes and environments. Here, in the present study, we have focused on three plant-height related traits (Ph: plant main stem height, Fbl: the first branch length, and Nsk: the number of nodes on the main stem) and had good control of the environmental factors during both experiments and data analyses. We find significantly associated SNPs for two of the studied traits (Ph and Fbl), KEGG and GO analyses of the genes from these regions together with those from twelve earlier QTL/GWAS studies for the same traits show that both Ph- and Fbl-associated genes are enriched in the biosynthesis of secondary metabolites. Secondary metabolites are usually specific to a narrow arrangement of species, defining the diverse “personalities” of different organisms, caffeine, alkaloids, flavonoids, and tannins represents good examples of secondary metabolites in plants [24]. Secondary metabolites are traditionally believed not to play key roles in plants’ primary life (like development, growth, and reproduction), however, they are well known for their enormous genetic and chemical diversity both across and within species, which definitely suggests the importance of their presence [24,25,26,27]. Popular speculation about secondary metabolites’ importance is their involvement in the ecological interactions of plants with the highly challenging and varying environments (both biotic and abiotic), which is also supported by many studies [26,28]. For example, when exposed to drought conditions, alkaloids production has been shown to be enhanced in plants for coping with the oxidative stress caused by water shortage [29,30]. Generally, under stresses, the elevated secondary metabolite production requires resources to be reallocated from other important activities such as growth [26,28,31]. Therefore, it may be not surprising for us to find secondary metabolite biosynthetic genes being highly represented within the genomic regions that are associated with peanut height-related traits, and this overrepresentation may suggest that it is common for peanuts to face intensive environmental stresses even under well-controlled experimental conditions.

Candidate genes for Ph and Fbl are also both highly represented in beta-Alanine metabolism. As the precursor of CoA, beta-alanine is important for the metabolisms of phospholipids and fatty acids, as well as for the tricarboxylic acid cycle. It has also been shown that beta-alanine can protect plants from a variety of abiotic and biotic stresses (such as extreme temperature, drought, and heavy metal) [19,32,33], perhaps, apart from the above-mentioned biotic stresses, globally elevated summer temperatures as a consequence of climate change may represent another source of stress for peanut. In addition, Ph- and Fbl-associated genes tend to be both involved in some basic and essential catalytic activities (hydrolase and oxidoreductase activities).

In addition to the above-mentioned common pathways/activities for Ph and Fbl, there are also trait-specific enrichments that are mostly in basic processes, pathways, or complexes, which are supposed to be crucial for plant development and growth. For example, Ph-associated genes are also enriched in brassinosteroids biosynthesis, ascorbate and aldarate metabolism, and regulation of epidermis development [33,34]. For Fbl, its candidate genes are also enriched in amino acid metabolism, ether metabolic process, amine biosynthetic process, RNA splicing, polyamine metabolic process, and water transmembrane transporter activity.

However, the interpretation of both GO and KEGG results need to be cautious, considering the fact that not all predicted peanuts proteins can be mapped to a known GO term or KO ID and it is common for one enzyme to be involved in several different pathways or processes [35]. Therefore, future investigation is needed to verify our findings.

## 4. Materials and Methods

### 4.1. Plant Materials

In this study, a total of 159 peanut accessions were collected from 29 Chinese provinces, representing the peanut cultivation areas in China. These accessions belonged to two subspecies and four botanical varieties: 89 *A. hypogaea* ssp. *hypogaea* (66 var. *hypogea* and 23 var. *hirsuta*), 61 *A. hypogaea* ssp. *fastigiata* (51 var. *vulgaris* and 10 var. *fastigiata*), and nine irregular types (Figure S1; Table S1). The irregular types usually were the hybrids among the four botanical varieties [23,36].

### 4.2. Phenotypic Statistics

All peanut accessions were planted at three different locations (Dongying, Juxian, and Laixi) in China during 2013, 2014, and 2016. Each accession was represented by 34–40 plants that were grown in a two-row plot (5.00 m long and 0.80 m wide). Three plant height-related traits were measured for each accession: main stem height (Ph), the number of nodes on the main stem (Nsk), and the first branch length (Fbl). Each trait for each harvested accession per location and per year was repeatedly measured five times. To minimize environmental effects, a mixed linear model was built for each trait (as response variable) using the R version 3.6.1 function “lem4” (https://www.r-project.org/, (accessed on 5 July 2019)). Within this model, all included explanatory variables had random effects: the nine combinations between growing years and locations were considered as environmental blocks, the five repeats for each studied plant accession were nested within a block, the peanut accessions, and the interaction between accession and environmental blocks. From the constructed model, the BLUP (Best Linear Unbiased Prediction) estimates of the random genetic effect of peanut accessions on the trait that was on focus were extracted, and it was these BLUP values that would be used (as response variable) later on in the GWAS analyses to study their associations with the identified high-quality SNPs (see below) [37]. The correlation coefficients of each pair of the analyzed traits were calculated with the R version 3.6.1 function “cor” (https://cran.r-project.org/bin/windows/base/, (accessed on 5 July 2019)), and the broad-sense heritability (*H^2^*) for each trait was estimated using the R package “lem4”.

### 4.3. Genome-Wide SNP Collection

High-quality NGS sequence reads acquired via a genotyping by sequencing (GBS) approach for the 159 studied peanut accessions were downloaded from the Sequence Read Archive database (SRA accession: PRJNA525244) [18]. The acquired high-quality reads were then mapped onto a peanut reference genome (https://www.peanutbase.org/data/public/Arachis_hypogaea/Tifrunner.gnm1.KYV3/ (accessed on 5 July 2019)) using BWA v0.6.2 (-t 4 –M –k 32 –r 1 –c 1) [38]. Variant calling was performed using the GATK’s Unified Genotyper (https://software.broadinstitute.org/gatk (accessed on 5 July 2019)). The identified variants were filtered to reduce the false-positive errors using GATK Variant Filtration. To im-prove variation quality, the following criteria were adopted: (i) quality score >20; (ii) coverage depth >3 fold; (iii) missing ratio within each population <80%; (iv) a global minor allele frequency (MAF) >0.05. The genic and intergenic locations of all high-quality variants (including SNPs) were determined using ANNOVAR [39].

### 4.4. Genome-Wide Association Study Analysis

Genome-wide association study (GWAS) of the three plant height-related traits based on the acquired high-quality SNPs were conducted using TASSEL v5.0 [37,38] Four different models were tried for each trait, including two general linear models: GLM, *Y* = *Xa* + *e*; GLM(G), *Y* = *Xa* + *Qb* + *e*, and two Mixed Linear Model: MLM(K), *Y* = *Xa* + *Ku* + *e*; MLM(GK), *Y* = *Xa* + *Qb* + *Ku* + *e*, where *Y* denoted the phenotype (represented by the BLUP values as estimated above) and *X* the genotype at each SNP locus, *Q* represented population structure estimated by Admixture and *K* was the relationship between samples (i.e. kinship coefficients). For each trait, the best model out of the tested four based on the Q-Q plot was accepted as the final model. We used a *p*-value of 10^−5^ or less to establish the significance. The candidate genome regions that might contain genes responsible for the studied plant height-related traits were the most strongly associated SNPs (hereafter referred to as peak SNPs) plus a 100kb-long genomic region that were centered on the peak SNPs [18,40,41,42]. The matrix of pairwise kinship coefficients among the studied accessions was calculated using the software SPAGeDi v1.5 [43].

### 4.5. A Literature Survey of QTLs for Peanut-Height Related Traits

To look for more candidate genes underlying peanut height-related traits, we carried out a thorough literature survey of related QTL/GWAS studies. All genes within the candidate genomic regions that were identified by both the present GWAS analyses and earlier studies were considered as candidate genes for the related traits. Both Gene Ontology (GO) enrichment analysis, and KEGG (Kyoto Encyclopedia of Genes and Genomes pathway database) pathway enrichment analysis was carried out on the candidate genes using the OmicShare tools, a free online platform for data analysis (www.omicshare.com/tools, (accessed on 5 July 2020)). To accommodate the multiple testing problem in the enrichment analysis, Benjamini–Hochberg adjusted *p*-values were calculated [44].

## Figures and Tables

**Table 1:** Phenotypic Statistics of the Three Studied Plant Height Related Traits.

Trait	Sample	Mean	SD	SE	Median	Min	Max	Range	Skew	Kurtosis	CV%	*H* ^2^
Fbl	1422	48.61386	13.4337	0.356243	48.2	11.6	87.8	76.2	0.22002	−0.35783	27.63	0.513899
Nsk	1423	17.47671	4.318244	0.114473	17.4	5.2	30.4	25.2	0.037926	−0.18738	24.71	0.443737
Ph	1417	35.078	10.65981	0.283181	34.0	6.6	67.8	61.2	0.450744	−0.11826	30.39	0.473302

NOTE: SD, standard deviation; SE, standard error; CV, coefficient of variance; *H*^2^, broad-sense heritability.

## Data Availability

Not applicable.

## Supplementary Materials

The following are available online at https://www.mdpi.com/article/10.3390/plants10061058/s1. **Figure S1**: Geographic distribution of the analyzed peanut accessions. Each accession is displayed as a dot. Different colors stand for the different botanical varieties. (NOTE: The map used here represents only part of China). **Figure** **S2**: The overall frequency distributions for the three studied plant height-related traits, included the main stem (Nsk), the first branch length (Fbl), and plant main stem height (Ph). **Figure S3**: The frequency distribution for the number of nodes on the main stem (Nsk) in different years at different locations. **Figure S4**: The frequency distribution for the first branch length (Fbl) in different years at different locations. **Figure S5**: The frequency distribution for plant main stem height (Ph) in different years at different locations. **Figure S6**: The correlation between the three studied plant height-related traits (Fbl, Ph, Nsk). **Figure S7:** Manhattan plots and Q-Q plots from the GWAS analyses of two plant height-related traits (Ph and Fbl). For each trait, the Q-Q plots from four different statistical models are shown on the right. The shown Manhattan plots are from the best model. The black arrows pointed to 11 peak SNPs that are associated with Ph and/or Fbl. The significance level is −log10 (0.05/17117) = 5.1 (the red horizontal line). **Table S1**: Peanut cultivars’ code numbers and sources. **Table S2**: A summary of the acquired SNPs and InDels in different genic and intergenic regions. **Table S3**: The correlation among the plant height-related traits. **Table S4**: Information on earlier identified QTLs for Ph and Fbl and their physical locations. **Table S5**: A summary of the specific/common candidate genomic regions for Ph and Fbl that are acquired from both the present and earlier studies. **Table S6**: Information on the annotated genes from the candidate genome regions for Ph and Fbl that are acquired from both the present and earlier studies.
