# Supplementary material for: The Genetic Base for Peanut Height-Related Traits Revealed by a Meta-Analysis
Source: Plants (Basel). 2021 May 25;10(6):1058. doi: 10.3390/plants10061058 (PMC8227209; doi:10.3390/plants10061058)
Supplement: Supplementary file 1 [file plants-10-01058-s001.zip › plants-1208579-supplementary/Supplementary_Figures.pdf]

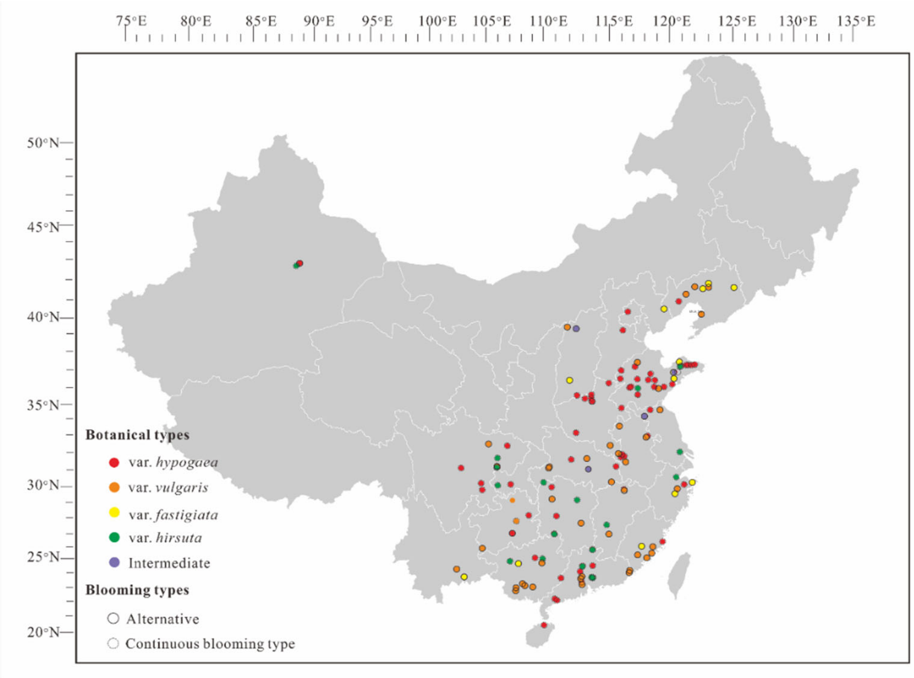

Figure S1. Geographic distribution of the analysed peanut accessions. Each dot represents an accession. Different botanical varieties are in different colours. (NOTE: The map used here represents only part of China).

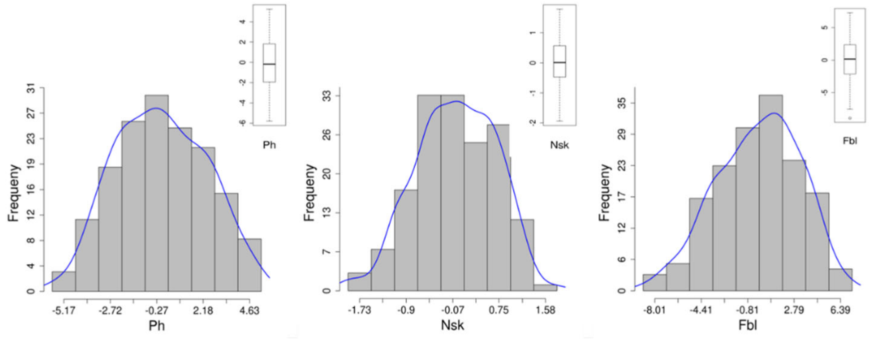

Figure S2. The overall frequency distributions for the three studied plant height-related traits.

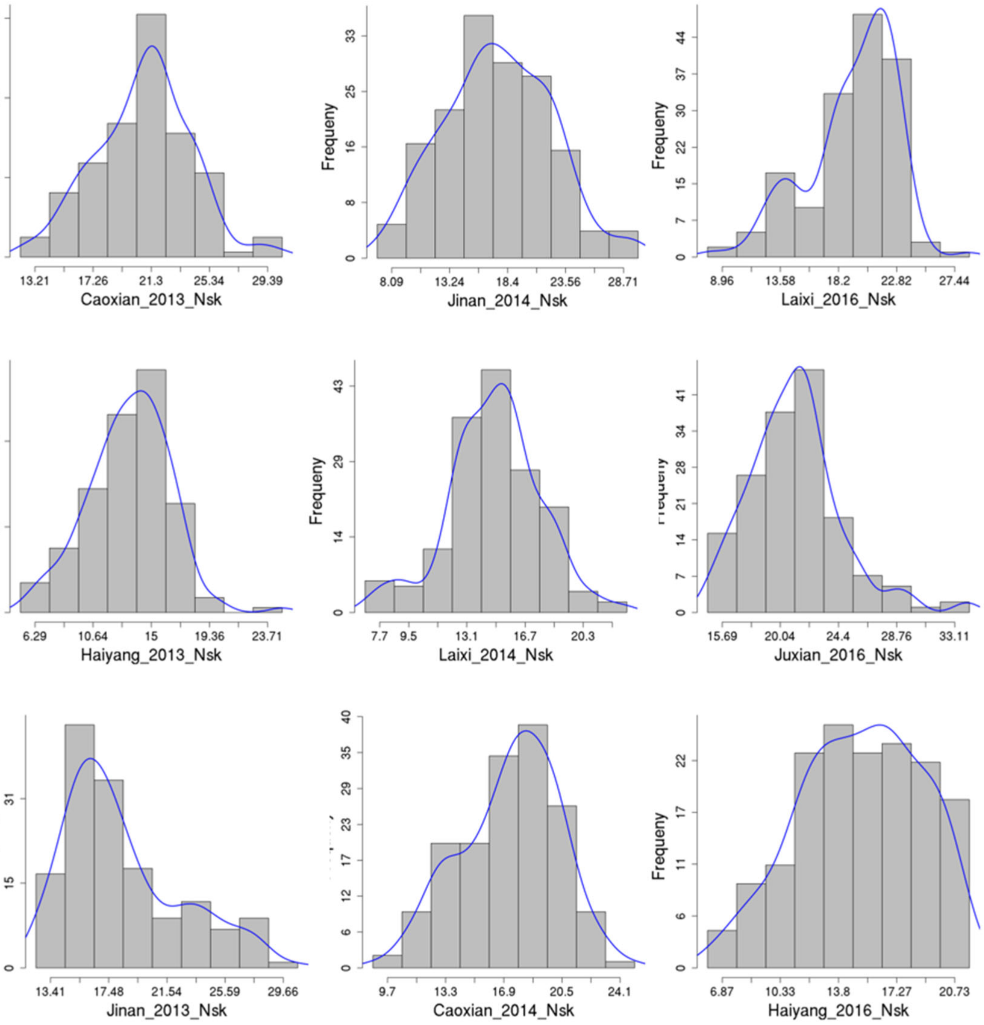

Figure S3. The frequency distribution for the number of nodes on the main stem (Nsk) in different years at different locations.

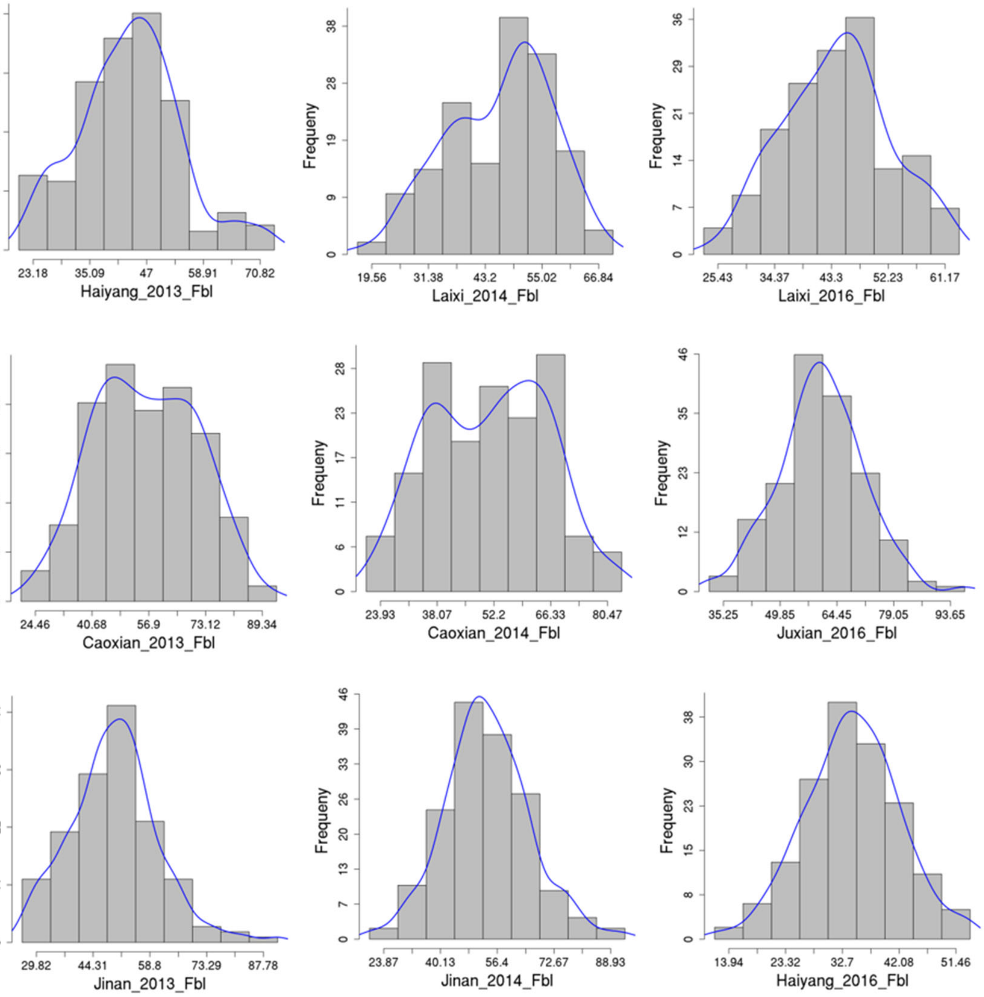

Figure S4. The frequency distribution for the first branch length (Fbl) in different years at different locations.

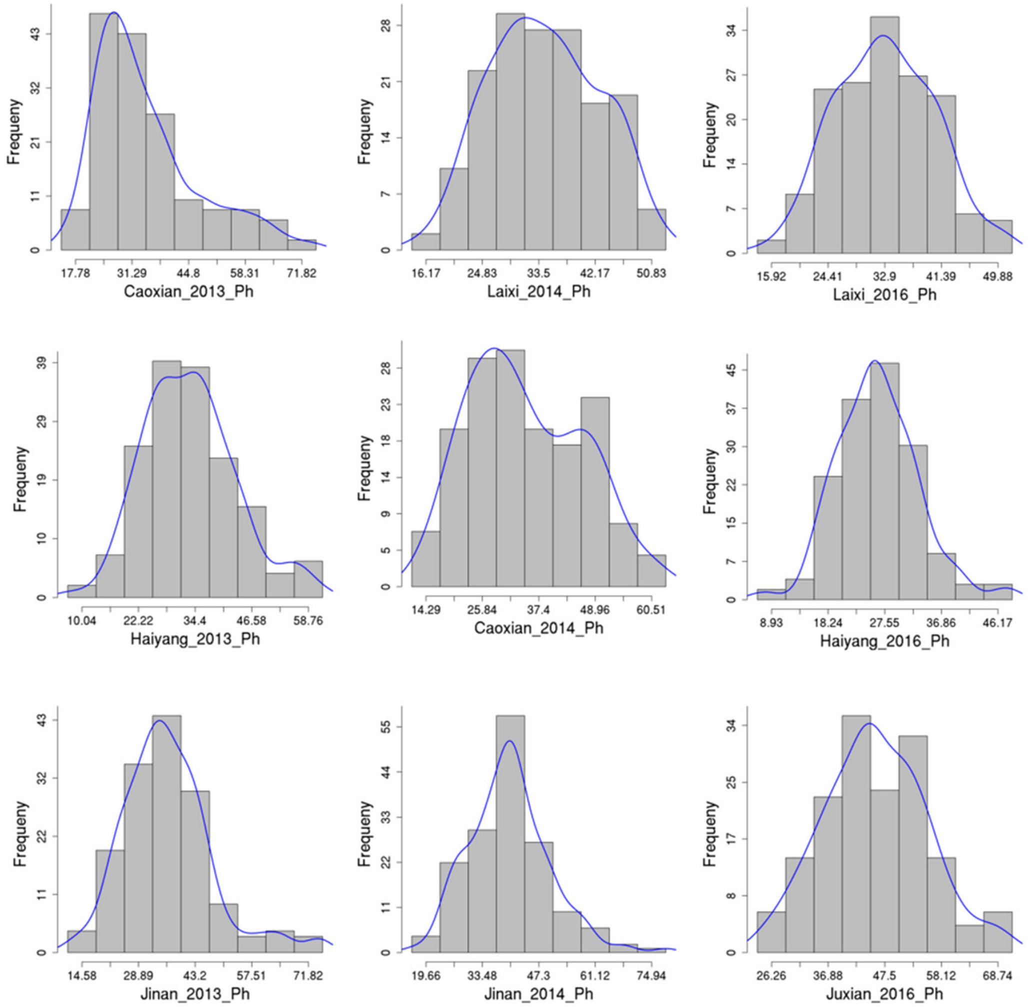

Figure S5. The frequency distribution for plant main stem height (Ph) in different years at different locations.

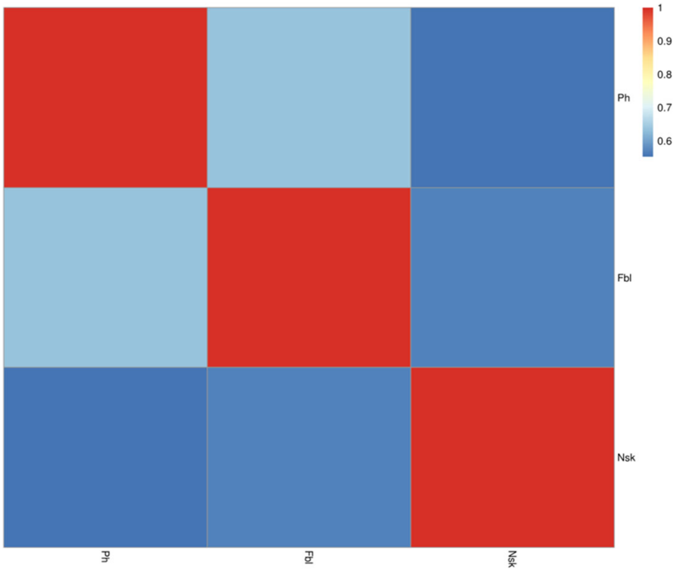

Figure S6 The correlation between the three studied plant height related traits (Fbl, Ph, Nsk).

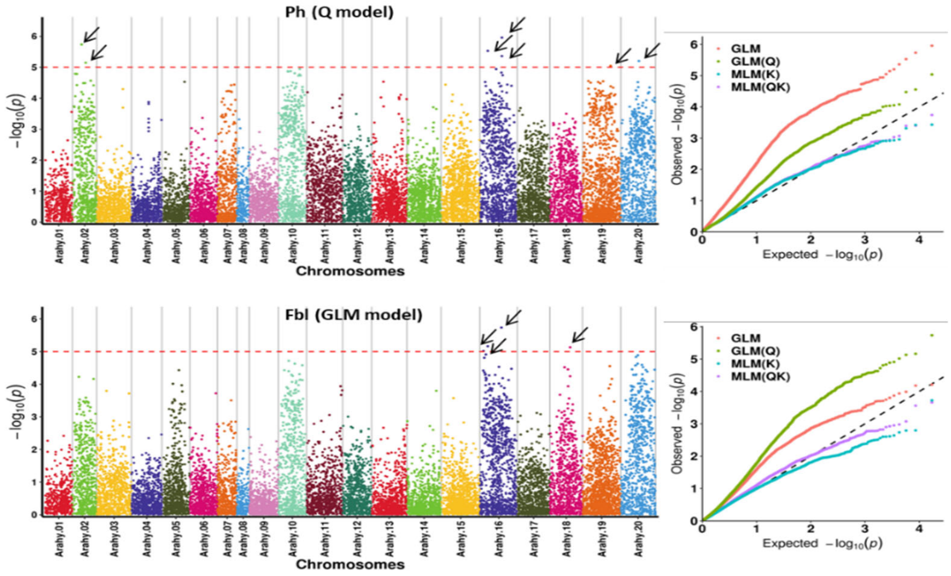

Figure S7. Manhattan plots and QQ plots from the GWAS analyses of two plant height-related traits (Ph and Fbl). For each trait, the QQ plots from four different statistical models are shown in the right. The shown Manhattan plots are from the best model. The black arrows pointed to 11 peak SNPs that are associated with Ph and/or Fbl. The significance level is $-\log_{10}(0.05/17117) = 5.1$ (the red horizontal line).
