# Supplementary material for: The Genetic Base for Peanut Height-Related Traits Revealed by a Meta-Analysis
Source: Plants (Basel). 2021 May 25;10(6):1058. doi: 10.3390/plants10061058 (PMC8227209; doi:10.3390/plants10061058)
Supplement: Supplementary file 1 [file plants-10-01058-s001.zip › plants-1208579-supplementary/Supplementary_Tables.pdf]

Table S2. A summary of the acquired SNPs and InDels in different genic and intergenic regions.

Type	InDels	Percentage	SNPs	Percentage
**Intergenic**	1,119	71.68%	14,019	90.12%
**Exonic**	79	5.06%	474	3.05%
**Intronic**	216	13.84%	588	3.78%
**Up-/ down-stream**	147	9.42%	475	3.05%
**Total**	1,561		15,556	

Table S3. The correlation among the plant height related traits.

	Ph	Fbl	Nsk
**Ph**	1	0.636356	0.552309
**Fbl**	0.636356	1	0.568581
**Nsk**	0.552309	0.568581	1
